# Oxidative Stress and NO Signalling in the Root Apex as an Early Response to Changes in Gravity Conditions

**DOI:** 10.1155/2014/834134

**Published:** 2014-08-17

**Authors:** Sergio Mugnai, Camilla Pandolfi, Elisa Masi, Elisa Azzarello, Emanuela Monetti, Diego Comparini, Boris Voigt, Dieter Volkmann, Stefano Mancuso

**Affiliations:** ^1^DISPAA, University of Florence, Viale delle Idee 30, 50019 Sesto Fiorentino, Italy; ^2^HSO-USB, ESTEC, European Space Agency, Keplerlaan 1, 2200 AG Noordwijk, The Netherlands; ^3^IZMB, University of Bonn, Kirschallee 1, 53115 Bonn, Germany

## Abstract

Oxygen influx showed an asymmetry in the transition zone of the root apex when roots were placed horizontally on ground. The influx increased only in the upper side, while no changes were detected in the division and in the elongation zone. Nitric oxide (NO) was also monitored after gravistimulation, revealing a sudden burst only in the transition zone. In order to confirm these results in real microgravity conditions, experiments have been set up by using parabolic flights and drop tower. The production of reactive oxygen species (ROS) was also monitored. Oxygen, NO, and ROS were continuously monitored during normal and hyper- and microgravity conditions in roots of maize seedlings. A distinct signal in oxygen and NO fluxes was clearly detected only in the apex zone during microgravity, with no significant changes in normal and in hypergravity conditions. The same results were obtained by ROS measurement. The detrimental effect of D'orenone, disrupting the polarised auxin transport, on the onset of the oxygen peaks during the microgravity period was also evaluated. Results indicates an active role of NO and ROS as messengers during the gravitropic response, with probable implications in the auxin redistribution.

## 1. Introduction

During evolution, plants have developed elaborate sensory and signaling systems to cope with and adjust to rapid environmental changes. Among them, gravity remains a constant stimulus, playing a central role in driving the evolution of plants on Earth [1]. Gravitropism involves a fine and reliable coordination of the activity of different cells and tissues deputed to gravity sensing with a growth response occurring in spatially distinct regions. In roots, for example, the centrally located columella cells in the root cap are the proposed site of gravity sensing, but the growth response (root curvature) occurs in the elongation zone (EZ). The most common and accepted explanation for gravity sensing in plants is the starch-statolith hypothesis (which is the physical sedimentation of starch-filled organelles called amyloplasts (statoliths) in gravity-sensing cells (statocytes) located at the root tip) which triggers biochemical and physiological signals [2]. After the first event (sensing the change in the gravity vector/level) a signal is transduced and then transmitted to the EZ, stimulating the differential cellular growth mentioned above. This response is mainly driven by auxin [3], which accumulates to higher levels along the lower side of the root, thus provoking the inhibition of growth (Cholodny-Went theory). Recently, other investigators highlighted the role of cytoskeleton in the gravitropic response [4, 5]. Its central role in modulating cell polarity, organelle movement, intracellular transport, and cell expansion leads cytoskeleton to be a strong candidate in mediating the gravity signal transduction cascade (tensegrity model, [6]).

In the last years, experimental evidences depicted the structure of the root apex as divided into three different zones: a transition zone (TZ) located between two other regions, the apical division zone (DZ), and the elongation zone (EZ) [7]. The cells belonging to TZ have a specific cytoarchitecture, with centralized postmitotic nuclei surrounded by perinuclear microtubules radiating toward the cell periphery. In contrast to the mitotically active DZ cells, which are continually assembling and disassembling mitotic spindles, the TZ cells are not deputed to perform these activities but to have more specific sensory activities [3]. Experimental data suggest that the TZ is more a sort of sensory and information processing zone, devoted to a continuous monitoring of the environmental parameters and triggering appropriate responses, rather than being implicated in the division and growth process. The TZ cells are very sensitive to a wide range of stress sources, such as touch [8], water and salt stress [9], aluminium [10], and hypoxia [11, 12]. However, little is known about the role of TZ cells in the graviresponse, especially related to the transduction/transmission phases after gravity sensing by the cells of the root tip.

Among key signalling molecules in plants, nitric oxide (NO) has recently emerged as an essential compound [13]. Among its tasks, NO regulates the actin cytoskeleton [14], endocytosis, vesicle trafficking, and the polarity of growing tip cells [15], root formation [16], and stomatal regulation [17]. In addition, NO is widely implicated in the plant response to environmental stress [18], but its exact role in the response of plants to change in gravity levels is still unclear and not well investigated.

Reactive oxygen species (ROS) such as H_2_O_2_ are generally considered to be toxic by-products of respiration. However, recent experiments suggest that the production of ROS should have an important and active role as components of intracellular and extracellular signalling [19]. In particular, H_2_O_2_ is starting to be accepted as a second messenger for signals generated by means of ROS because of its relatively long life and high permeability across membranes [20]. The role of ROS in the gravitropic response is still under debate, as this topic has been rarely investigated [21].

Elucidating the mechanisms behind the signal transmission from the site of gravity sensing to the site of gravibending is therefore the main objective of this paper, with particular interest towards the role of the root apex (and in particular the TZ) in the transduction process and the importance of sensing molecules, such as NO and H_2_O_2_, in the gravity response process. After preliminary experiments on ground using the method of gravistimulation via a horizontal displacement of the root, the response of maize seedlings to gravity changes has been studied for the first time ever in a real situation of microgravity, thanks to a set of experiments performed during three ESA parabolic flight campaigns and a drop tower campaign.

## 2. Materials and Methods

### 2.1. Gravistimulation on Ground

Oxygen flux measurements were performed using the vibrating probe technique [22]. Briefly, healthy* Z. mays* L. root apices (5-6 mm long) were cut, carefully washed with deionized water, and placed individually at the bottom of a measuring chamber containing an electrophysiological solution (10 mM CaCl_2_, pH 6.5). The flux measurement was performed at 24 ± 0.25°C by positioning a custom-built oxygen-selective microelectrode (tip diameter of 1 *μ*m [23, 24]) near the root surface. To ensure the flux detection on the bottom part of a gravistimulated root (gravistimulation was performed by rotating the seedling and the measurement system by 90 degrees) an electrode with a hooked tip was also built. During the recording, the microelectrode oscillated in a square wave parallel to the electrode axis over a distance of 10 *μ*m (0.1 Hz frequency), moving along the entire root length. The calculation of the difference between the voltage of each electrode position and that of the previous one at the other extreme position as well as the evaluation of a moving average of these differences over any desired time period, producing the potential difference, were computer generated. The O_2_ influxes were calculated using Fick's first law of diffusion, assuming a cylindrical diffusion geometry. The flux measurements were performed on at least 10 different root apices per treatment (*n* ≥ 10).

To localize the production of NO in the different regions of the root apex with a spatial resolution of a few micrometers, a NO-selective microelectrode of carbon fibers with diameters as small as 5 *μ*m was constructed [12], using the same system described above for the measurement. The dimensions and the response time (<0.5 s) allowed the use of this electrode in a self-referencing mode [23, 25] with a resolution as small as 50 fmol cm^−2^ s^−1^.

### 2.2. Parabolic Flight Experiments

All parabolic flight experiments were conducted aboard the Airbus A300 ZERO-G, which is operated by Novespace and is based in Bordeaux, France. Every parabolic flight, which lasts ~3 h including takeoff and landing, encompasses 31 parabolas. Every parabola started from a steady normal horizontal flight and typically included 2 hypergravity (1.8 g) periods of 20 s, separated on average by a 22-s microgravity period (<0.05 g). The first test parabola was followed by 6 series of 5 parabolas, separated by breaks of 4 and 8 min, respectively. The data presented emerged from the 41st and 45th Parabolic Flight Campaigns (PFC) and the “Fly Your Thesis 2012” (student campaign) of the European Space Agency (ESA), representing a total of 8 parabolic flights or 248 parabolas.

During the 41st ESA Parabolic Flight Campaign (PFC), the measurements of oxygen influx/efflux from the seedling roots were conducted. For each parabolic flight of the PFC, a set of three 3-day-old seedlings of* Zea mays* L., with a homogeneous length of 5 ± 0.5 cm, have been installed into an Eppendorf vial (1 seedling = 1 vial) filled with an electrophysiological solution (10 mM CaCl_2_, pH 6.5). The fourth vial was left empty and used as a control without seedlings. A couple of O_2_ needle microsensors (OX50, Unisense, DK) have been horizontally inserted at two different levels for each vial, corresponding to the root apex and to the mature zone of the seedling root. The tip of the electrode was placed close to the root tissues (distance < 1 mm). The vials with the seedlings and the electrodes were placed inside a thermostated chamber (temperature = 24-25°C). Each electrode was connected to a picoammeter (PA2000, Unisense, DK), a four-channel laboratory amplifier that enables the measurement of multiple parameters. The output of the picoammeter was then connected to a datalogger. A dedicated LabView software on a laptop recorded the oxygen measurement. Concurrently, an accelerometer provided gravity measurement.

During the 45th ESA PFC the respiration rate of detached root apices (*n* = 6, with known weight) from 3-day-old* Zea mays* L. seedlings was measured. The root apices were placed inside an oximeter chamber (Oxytherm, Hansatech Instruments) with controlled temperature (25°C). A small magnetic stirrer provided a continuous stirring of the solution. Measurements have been performed with the apices in distilled water or in a solution containing D'orenone (C_18_ ketone (5*E*,7*E*)-6-methyl-8-(2,6,6-trimethylcyclohex-1-enyl) octa-5,7-dien-2-one, 10 *μ*g/mL). The oximeter chamber was connected to a laptop with a dedicated software for datalogging.

During the “Fly your thesis 2012” PFC campaign the production of H_2_O_2_ was assessed. The Amplex Red reagent was used after a preliminary evaluation in our lab, due to its high sensitivity and successful use for the measure of H_2_O_2_ production in plant root, as previously reported by [26]. We used the Amplex Red Hydrogen Peroxide/Peroxidase Assay Kit (Invitrogen, #A22188) in combination with horseradish peroxidase (HRP, Invitrogen), to detect H_2_O_2_ released from biological samples. In the presence of HRP Amplex red reagent reacts with H_2_O_2_ in a 1 : 1 stoichiometry to produce the red-fluorescent oxidation product, resorufin. As first step, the tips of maize roots were cut and immediately washed twice for 15 minutes in PBS to eliminate ROS derived from the cut. Tips were divided in samples constituted by 10 mg of fresh tissue. Then 50 *μ*L of working solution (containing dye and HRP) was added to 10 mg of plant tissue (root), and the samples were incubated in a 96-well microplate at room temperature for 30 minutes, in darkness. During the flight, fluorescence was measured using excitation at 530 ± 12.5 nm and fluorescence detection at 590 ± 17.5 nm by using a microplate reader (Tecan Infinite 200 PRO). A datalogger connected to the Tecan and a laptop provided data storage, which were then normalised to plant biomass.

### 2.3. Drop Tower Campaign

The Drop Tower in Bremen (Germany) is one of only a few facilities worldwide providing gravitational forces as small as 10^−5 ^g, even if only for a short time of 4.7 seconds. The cylindrical falling capsule of a diameter of 80 cm, a height of 2.8 m, and a mass of 500 kg is dropped from 110 m height of the tower whose inner tube is evacuated within 2 h to an air pressure of less than 10 mPa. On the bottom of the tower the capsule (reaching 170 km h^−1^) is decelerated within 130 ms by a huge basket of app. 2.5 × 8 m, filled with styropor grains. There the motion energy (6 × 10^5^ Nm) is converted into heat. Gravitational sensors were provided by ZARM. Deceleration of the capsule leads to gravitational values of about 30 g. An oximeter (see Parabolic Flights section) was used and adapted for the measurement of nitric oxide by using selective microelectrodes (amiNO-30, Innovative Instruments Inc., USA) connected to a NO electrochemical detector with automatic temperature compensation (in NO-T-II, Innovative Instruments Inc., USA). The output of the detector was connected to a laptop via USB port running the inNO-T-II specific software for data acquisition.

## 3. Results 

### 3.1. Gravistimulation on Ground

**Figure 1:**
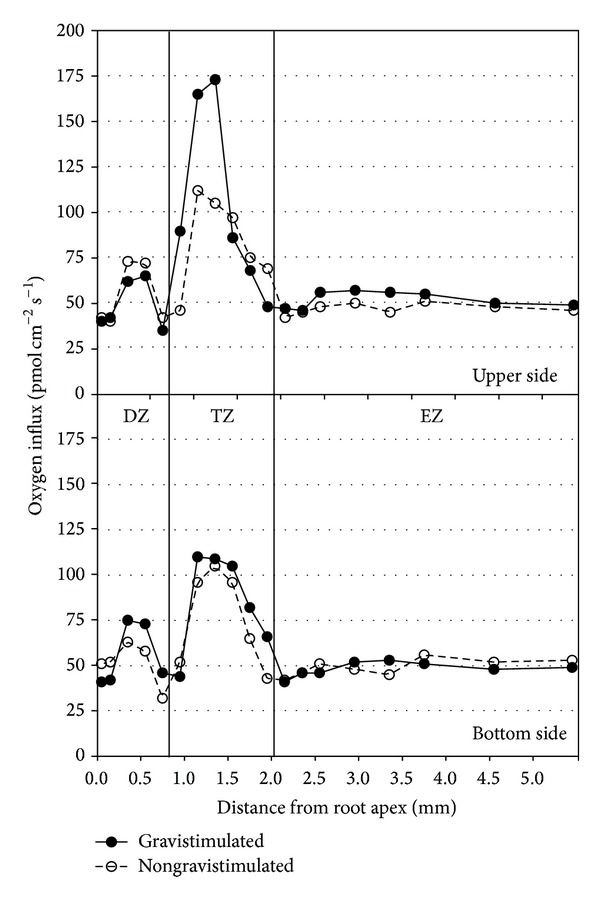
Oxygen influx measured on the two sides of a gravistimulated and a vertical root. Upper graph refers to the upper side of a gravistimulated root, whereas the second graph refers to the bottom side of a gravistimulated root.

**Figure 2:**
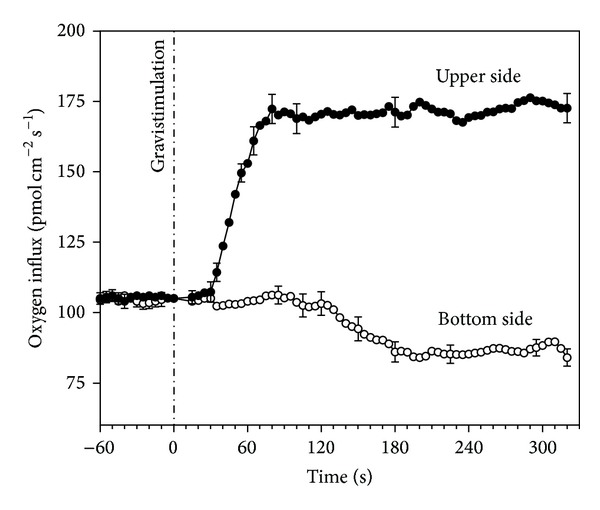
Timeline of the oxygen influx change at TZ level after gravistimulation (time = 0).

In normal (vertical) conditions, strong differences between the constituent zones of the maize root (DZ, TZ, and EZ) were clearly evident. The TZ appeared to be the most active zone in the uptake of oxygen from the surrounding solution (Figure 1). The spatial patterns of the oxygen influxes in the entire root apex showed a marked peak in the TZ (110 pmol cm^−2^ s^−1^) at 1–1.5 mm from the maize root tip. A minor oxygen influx peak (75 pmol cm^−2^ s^−1^) was also evident in the DZ. Importantly, the TZ was the only root apex region significantly affected with regards to gravistimulation; in fact, the marked peak of oxygen influx was greatly enhanced in the upper part of the horizontal (gravistimulated) root, whereas the DZ maintained a similar pattern. On the contrary, the bottom side of the horizontal root showed a normal behaviour. The increase of oxygen influx at TZ level appeared as a very quick response following gravistimulation, as it was clearly evident after less than 30 seconds in the upper side of the root (Figure 2), while the bottom part remained unaffected.

**Figure 3:**
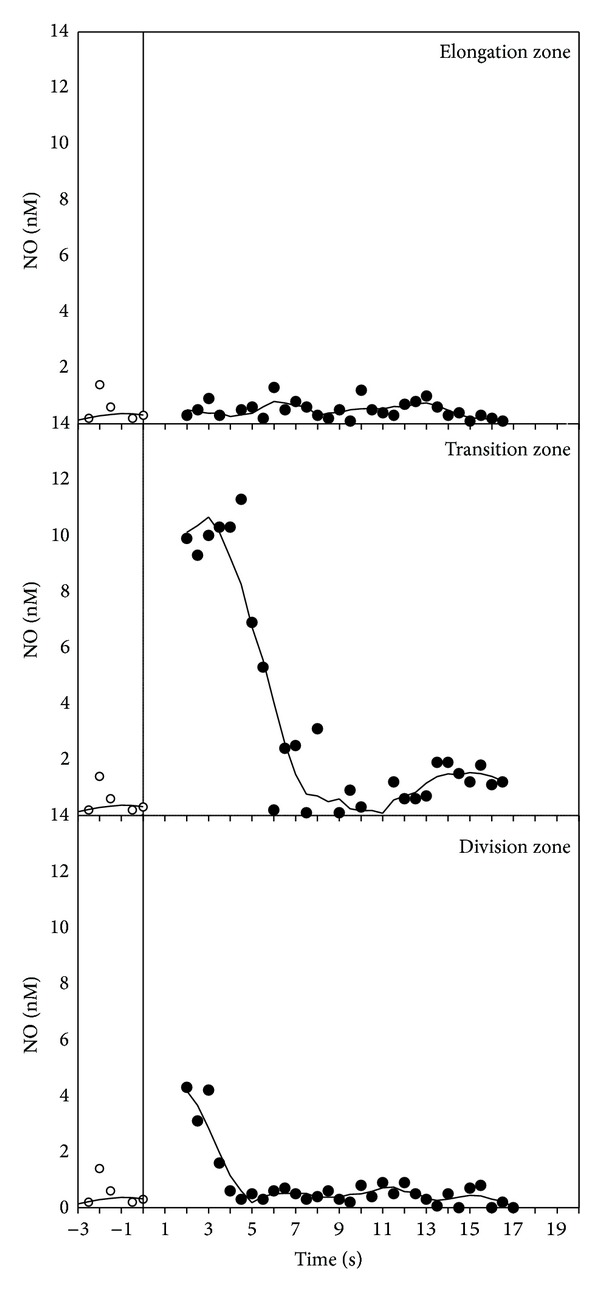
Timeline of the NO production in a maize root apex in the three different constituent zones after gravistimulation (time = 0).

Gravistimulation also promoted a very fast NO production from the root apex (Figure 3). A burst of NO was suddenly produced after only 2-3 seconds from gravistimulation, reaching a peak of 10 nM and lasting approximately 8 s before returning to the steady-state values in the TZ. Only a small and negligible efflux of NO was detectable in the DZ, and, importantly, NO bursts were not detected in the EZ region.

### 3.2. Parabolic Flight and Drop Tower Campaigns

**Figure 4:**
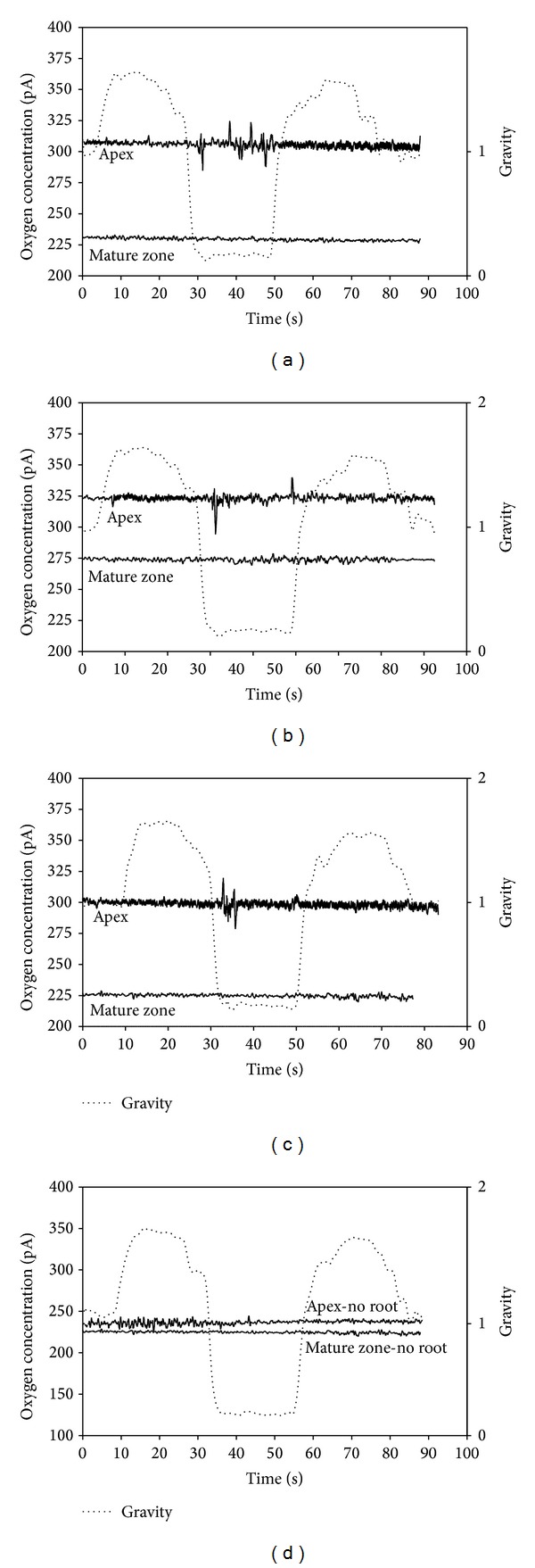
Oxygen concentration in the solution measured at two different levels, close to (distance < 1 mm) the root apex and to the root mature zone during three different parabolas: one parabola at the beginning (a), one in the middle (b), and one at the end of the experiment (c). Control experiment with no roots inside the Eppendorf vial is shown in (d).

A typical parabola is shown in Figure 4, with a period of microgravity (<0.05 g, 20–22 secs) inserted between two periods of hypergravity (1.8 g, 20 secs). Oxygen concentrations in the solution measured at two different root levels (root apex and mature zone) are also reported for three different parabolas. Bursts and peaks of oxygen concentration are clearly evident at root apex level during the microgravity periods, with significant detection during the entire flight. Interestingly, no peaks were detected in the mature zone and during hypergravity periods in the root apex. Control without seedlings showed no activity, demonstrating that the results previously shown were not related to a background noise of the microelectrodes during microgravity.

**Figure 5:**
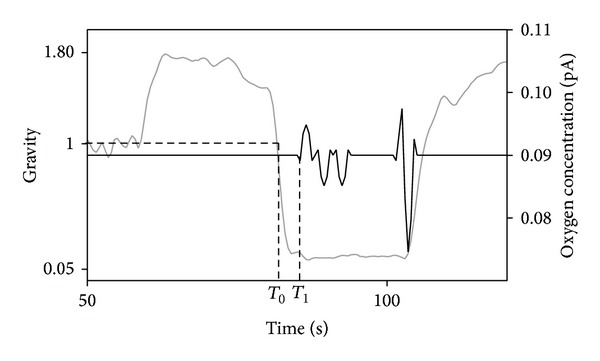
Timeline of burst appearance (*T*_1_) after the onset of microgravity (*T*_0_) during a parabola.

For each parabola, the average detection time (*T*_1_) of the first oxygen peaks from the start of the microgravity period (*g* < 1, *T*_0_; see Figure 5) has been calculated. The average time for all the parabolas is 2.4 secs. In order to evaluate a difference in the appearance of the first peak during the flight, the parabolas have been separated in three different groups (Table 1). Each group was composed of 10 parabolas. The objective was to investigate if a sort of “memory effect” of the stimulus during the repeated parabolas could cause a different response during the time of the flight (higher/lower, anticipated/retarded). The results showed a reduction, but not statistically significant, in the onset of the first peak during the last set of parabolas.

**Figure 6:**
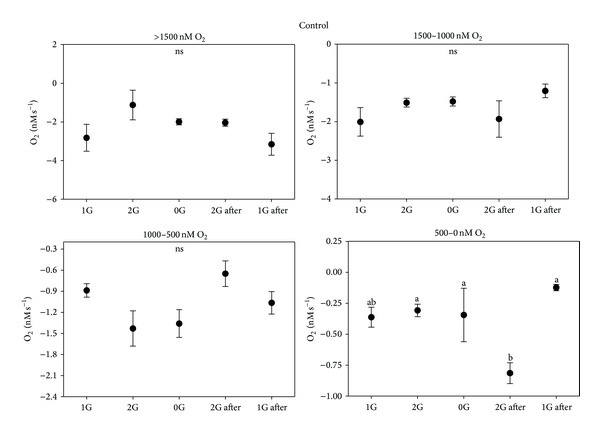
Respiration rate in control roots for the different groups related to the oxygen concentration in the solution.

**Figure 7:**
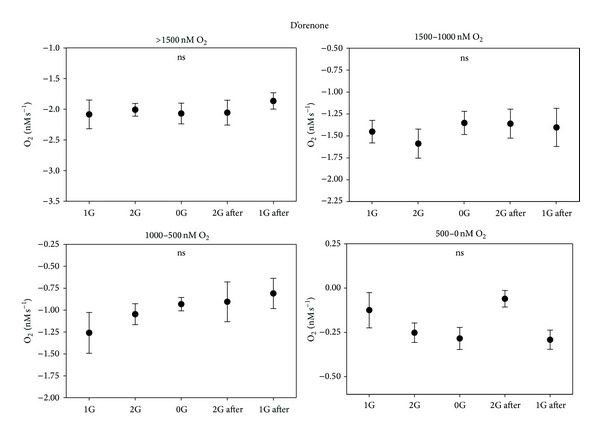
Respiration rate in roots incubated in D'orenone for the different groups related to the oxygen concentration in the solution.

**Figure 8:**
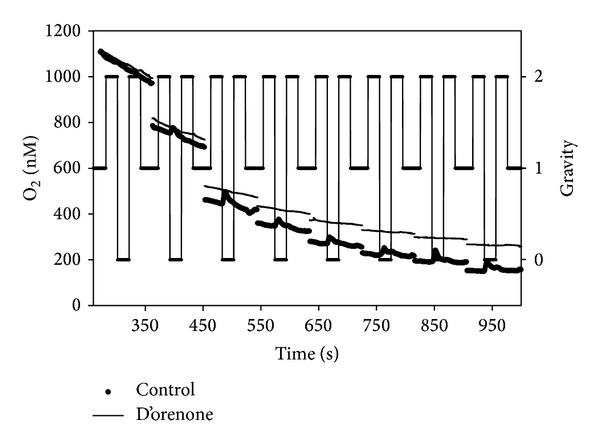
Respiration rate during single parabolas when [O_2_] in the solution was <700 nM. Both control roots and roots incubated in D'orenone are reported.

**Figure 9:**
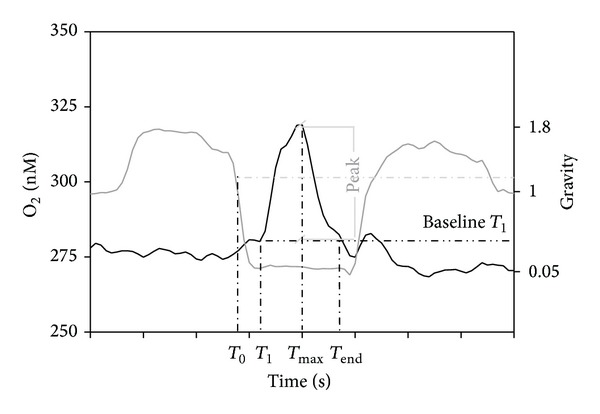
Characterisation of an oxygen burst measured with an oximeter: *T*_0_ is the time of the onset of microgravity; *T*_1_ is the time when the burst of oxygen begins. Its value in the *Y*-axis is taken as the baseline for the calculation of the area, amplitude, and duration between *T*_1_ and *T*_end_.

**Figure 10:**
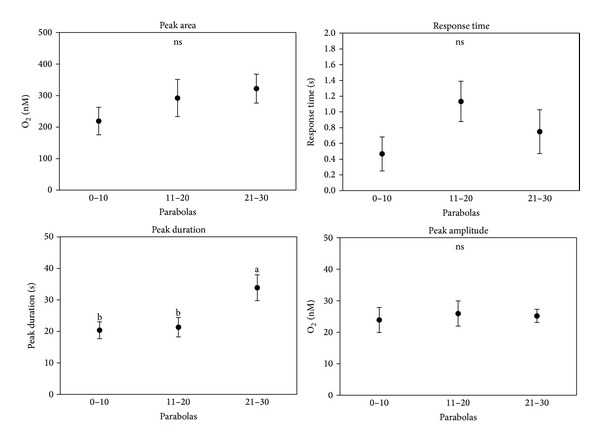
Peak area, response time, peak duration, and peak amplitude measured for different groups of parabolas by using the method described in Figure 9.

Interesting results have been also obtained from the measurement of the respiration rate by oximeters. The respiration rate inside a single parabola has been divided into five segments, each segment being related to a different gravity level: 1 g, 2 g, 0 g, 2 g (after microgravity), and 1 g (after microgravity). Negative values indicate oxygen influx. Respiration by root apices led to an unavoidable reduction of the oxygen content in the solution due to the plant metabolism; thus the parabolas have been divided into different groups by taking into account the real oxygen concentration because the respiration rate is directly related to the amount of oxygen present in the solution. Four groups related to different [O_2_] in the solution have been therefore created: >1500 nM, 1000–1500 nM, 500–1000 nM, and <500 nM. The values relative to the control (Figure 6) show no significant differences among the different gravity levels in each parabola for every oxygen concentration group, except for the last group ([O_2_] < 500 nM) with an increased respiration rate during the second period of hypergravity. On the contrary, the presence of D'orenone in the solution did not lead to any variation neither in the respiration rate among the different gravity levels nor compared to the control (Figure 7). D'orenone has been utilized in this experiment because it increases PIN2 protein abundance without affecting PIN2 transcripts, with the consequence that the PIN2 expression domain enlarges and shifts basipetally, resulting in more active auxin transport. To deeply analyse the previous results, the behaviour of the respiration rate during a single parabola has been evaluated. It has been noted that when [O_2_] was < 700 nM, a sudden burst of oxygen was produced only in the control a few seconds after the onset of microgravity (Figure 8). This large amount of oxygen was quickly absorbed by the roots for respiration, thus explaining the increased respiration rate during the second hypergravity period. This phenomenon was clearly evident during each parabola with [O_2_] < 700 nM. The fact that the bursts were evident only when [O_2_] < 700 nM was probably due to the electrode sensitivity, which was not able to discriminate very low differences in the respiration rate (around 25 nM) with higher oxygen concentrations in the solution. These oxygen bursts have been characterised by calculating the area inside the curve (Figure 9). The values of area, response time after the onset of microgravity, peak duration, and peak amplitude are reported in Figure 10, with a discrimination based on the parabolas' groups. No significant differences among the groups were noted in the peak area, with an average value of 274.73 nM representing the moles of oxygen produced during the microgravity period and then consumed, in the response time after the onset of microgravity (average value of 0.79 seconds) and the timing of the maximum peak (11.03 seconds). On the contrary, significant differences among groups were registered in the peak duration. The first 20 parabolas had an average peak duration of 20-21 seconds, while the last 10 parabolas had a longer duration (average value around 30 seconds). Finally, peak amplitude showed no significant differences. As expected, when no roots were present in the oximeter, a stable signal was registered (data not shown).

**Figure 11:**
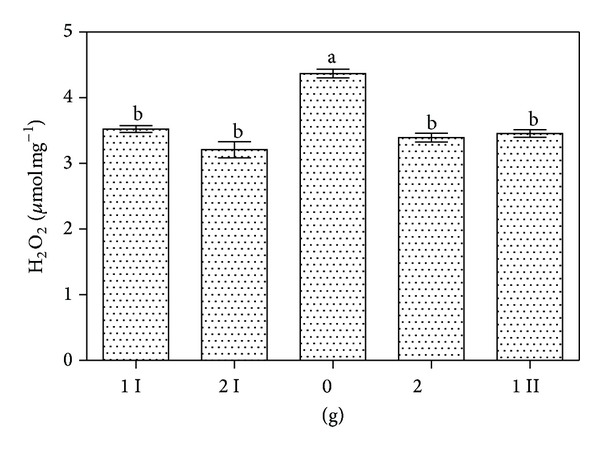
H_2_O_2_ production measured with Amplex Red during different gravity conditions. The letters I and II refer to the 1 g and 2 g phases prior (I) and after (II) the 0 g condition. Data are presented as average among each parabola of two parabolic flight days (62 parabolas in total are considered). Data were analyzed by ANOVA. Data statistically different are indicated with different letters (*P* < 0.05). Error bars are also indicated.

Production of H_2_O_2_ measured with Amplex Red is shown in Figure 11. Data were grouped according to the class of gravity level. Data recorded during microgravity (0 g) were statistically compared with data recorded during normogravity (1 g) and hypergravity (2 g) conditions. Transition from 1 g to 2 g and 2 g to 1 g had no significant effect on H_2_O_2_. On the contrary, transition from 2 g to 0 g results stimulated a higher H_2_O_2_ production from the root samples. Interestingly, we did not observe any difference in H_2_O_2_ production between 2 g condition in comparison to 1 g. Control experiment without any root tip inside the microplate showed no significant difference between all the gravity levels (data not shown).

**Figure 12:**
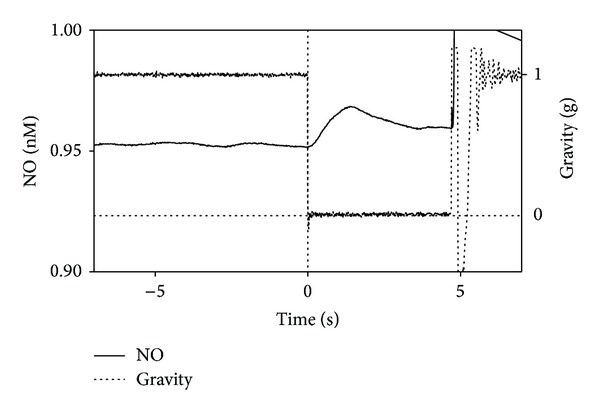
Typical nitric oxide curve measured in the oximeter chamber during a drop in the ESA Drop Tower campaign.

Finally, the production of NO was detected and evaluated during an ESA Drop Tower campaign (Figure 12). Interestingly, a burst of NO was clearly evident after 2 seconds from the start of the microgravity period, which then started to decline, resembling the behaviour of the gravistimulated roots on ground. Oximeter chambers without roots showed no bursts or signal detected by the microelectrode (data not shown).

## 4. Discussion 

Although both sensing (Cholodny-Went theory and tensegrity model) and signal transduction (role of auxin in the root bending) in the gravitropic response are well and comprehensively described in the literature, little is known about the grey area of signal transmission, the series of events comprised between sensing and bending. Hu et al. [27] reported that gravistimulation induced the asymmetric accumulation of nitric oxide (NO) on the lower side of the apical region of gravistimulated (horizontal) soybean seedling roots, leading to a subsequent auxin accumulation in the upper part. Our results confirmed this hypothesis, with a massive production of NO in a very short time (2-3 seconds). Moreover, we also integrated these results with the interesting information that the NO is mainly produced at TZ level, thus confirming the role of TZ as a sensing zone of the root, directly and actively implicated in the response to gravity changes.

Gravistimulation also induced a sudden burst of oxygen in the upper part of TZ level 20–30 seconds after gravistimulation. Our results suggest the hypothesis that after the displacement of statolythes under gravistimulation, the chain of events is related to a sudden emission of NO which leads to an improved plant metabolism which needs more oxygen for respiration, especially at TZ level, to produce ATP to be used as a source of energy. Rapid changes in cytosolic Ca^2+^ and pH have been proposed as components of the gravisignaling machinery [28]; therefore it is plausible that the control of Ca^2+^ and H^+^ channels would require more ATP (i.e., more oxygen consumption) after gravistimulation and during microgravity. The fact that oxygen burst at TZ level following gravistimulation can be inhibited by BFA [3] and that TZ shows significant higher auxin secretion via the endocytic vesicle recycling [29] might correlate the oxygen bursts observed during gravistimulation/under microgravity and the auxin metabolism, thus provoking a differential growth response.

In plants, the simultaneous generation of O_2_ and NO has a synergistic function in defense responses [30], as well as in plants exposed to abiotic stress [31]. NO is also generated at the same time as ROS, such as hydrogen peroxide, for example, during abiotic stress [32]. Root gravitropism appears to be another example of a physiological process in which both NO and ROS play key roles in a simultaneous way [33], as ROS were recently associated with auxin-mediated gravitropic responses in maize [21] and in the graviresponsive pulvinus of maize [34]. In gravistimulated roots, ROS accumulated asymmetrically to the lower cortex within 30 min of reorientation, becoming symmetrical upon longer stimulation [21]. Interestingly, Long et al. [35] have shown that auxin asymmetries are detectable only after 2 h of gravistimulation of the pulvinus, making the ROS changes reported much faster than the generation of gradients in auxin and so, in contrast to the conclusions from the gravitropically responding root, possibly placing them upstream of the action of this hormone. Our results support this hypothesis, with the generation of an oxygen burst after a few seconds after the onset of microgravity, which could be directly linked to the production of ROS as a stress messenger. The fact that the respiration rate in root apices increased during microgravity could also be related to the necessity of activating defensive and scavenging mechanisms for ROS molecules. In fact, the production of ROS during real microgravity has been confirmed during a parabolic flight campaign.

D'orenone rapidly and significantly activates the DR5 promoter [36] and also targets processes that are related to PIN2 degradation [16], causing slower turnover and increased protein levels of this auxin efflux transporter, thus suggesting that this apocarotenoid interacts with auxin signaling at the root apex. Our results indicate that D'orenone has also an inhibitor activity on the respiration rate and on the oxygen production, thus giving indirect clue to a link between the sudden increase of oxygen during microgravity and auxin redistribution via PIN2 activity which is one of the major responses to changes in the gravity vector/levels.

## 5. Conclusions

For the first time ever, a systemic and comprehensive series of experiments concerning the role of oxygen and stress messengers (NO and ROS) during a real microgravity environment has been conducted. The timeline and the cascade of events detected during these experiments suggest an active role of NO and ROS during the transmission step of the gravity response, with probable implications in the auxin redistribution.

## Figures and Tables

**Table 1:** Average time of burst appearance calculated for different groups of parabolas. Data were analyzed by ANOVA, using Tukey's test (*P* < 0.05).

Group of parabolas	Average time (secs)	Standard deviation	Significance
1–10	2.51	1.08	ns
11–20	2.65	1.22	ns
21–30	2.07	0.99	ns
1–30	2.4	1.1	—
